# Routine Clinical-Pathologic Correlation of Pigmented Skin Tumors Can Influence Patient Management

**DOI:** 10.1371/journal.pone.0136031

**Published:** 2015-09-01

**Authors:** Caterina Longo, Simonetta Piana, Aimilios Lallas, Elvira Moscarella, Mara Lombardi, Margherita Raucci, Giovanni Pellacani, Giuseppe Argenziano

**Affiliations:** 1 Dermatology and Skin Cancer Unit, Arcispedale Santa Maria Nuova-IRCCS, Reggio Emilia, Italy; 2 Pathology Unit, Arcispedale Santa Maria Nuova-IRCCS, Reggio Emilia, Italy; 3 Dermatology Unit, University of Modena and Reggio Emilia, Modena, Italy; The University of Queensland, AUSTRALIA

## Abstract

**Background:**

Several studies have demonstrated the benefit of integrating clinical with pathologic information, to obtain a confident diagnosis for melanocytic tumors. However, all those studies were conducted retrospectively and no data are currently available about the role of a clinical-pathologic correlation approach on a daily basis in clinical practice.

**Aim of the Study:**

In our study, we evaluated the impact of a routine clinical-pathologic correlation approach for difficult skin tumors seen over 3 years in a tertiary referral center.

**Results:**

Interestingly, a re-appraisal was requested for 158 out of 2015 (7.7%) excised lesions because clinical-pathologic correlation was missing. Of note, in 0.6% of them (13 out of 2045) the first histologic diagnosis was revised in the light of clinical information that assisted the Pathologist to re-evaluate the histopathologic findings that might be bland or inconspicuous *per se*.

**Conclusion:**

In conclusion, our study demonstrated that an integrated approach involving clinicians and pathologists allows improving management of selected patients by shifting from a simply disease-focused management (melanoma *versus* nevus) to a patient-centered approach.

## Introduction

Histopathologic examination is the gold standard for the diagnosis of skin tumors. In the typical scenario, a dermatologist or another clinician identifies a lesion as sufficiently atypical to warrant biopsy; then a pathologist provides the ultimate diagnosis, and finally the patient is managed accordingly. In a good proportion of cases the histopathologic morphologic features are straightforward and allow a confident diagnostic conclusion. However, for a small percentage of difficult melanocytic lesions, interpretation is rather subjective and the distinction between nevi and melanoma may be challenging, even for experienced pathologists. This is the case when the clinical information might help the pathologist to increase the diagnostic confidence.

Usually the clinical information provided to the pathologist is limited to the essential demographic data (age, gender, and body site of the lesion). However, there is growing evidence that providing clinical and dermoscopic images to the pathologist have the potential to improve his/her diagnostic confidence [1–12]. A number of previous studies have indeed demonstrated the benefit of integrating clinical with pathologic information, not only in the field of inflammatory skin disease but also in the context of skin tumors [2,3,6,7,9,10]. However, all those studies were conducted retrospectively and no data are currently available about the role of a clinical-pathologic correlation approach on a daily basis in clinical practice.

In this study, we evaluated the impact of a routine clinical-pathologic correlation approach for difficult skin tumors seen over 3 years in a tertiary referral center.

## Materials and Methods

This study was conducted from 2011 to 2013 at a tertiary referral center in Italy. Ethical approval was waived. First and senior authors were directly involved in patient care and had access to patient data prior anonymization. Senior Author was responsible of the anonymization of the patient data. Routine care was not altered and no additional data were collected for the specific purpose of this study. Patients were informed that their data would be used for research purpose and we collected written informed consent. The Local ethics committee from "Ethical Committee of the Province of Reggio Emilia, Italy" granted a waiver of approval because this study reflects our routine activity in managing patients with skin cancer. As a standard of care, all atypical skin tumors, identified by clinical-dermoscopic examination and thus scheduled for biopsy, are routinely imaged and stored in a dedicated database (DermoSun, Naples, Italy). More specifically, for each case a clinical overview, a close-up with a ruler and one dermoscopic image are collected. Clinical images are acquired using a Canon G15 (Canon Inc., Tokio, Japan) and dermoscopic images using dedicated lens (Dermlite Foto, 3Gen Inc, Dana Point, California, USA) at 10-fold magnification. Before excision lesions are usually examined by reflectance confocal microscopy (RCM), (Vivascope 1500, Mavig, Germany), whereas patients with multiple nevi undergo sequential digital dermoscopy imaging using a dedicated equipment (Molemax HD, DermaInstrument, Vienna, Austria). Changing lesions are excised and images captured with the system mentioned above.

**Fig 1:**
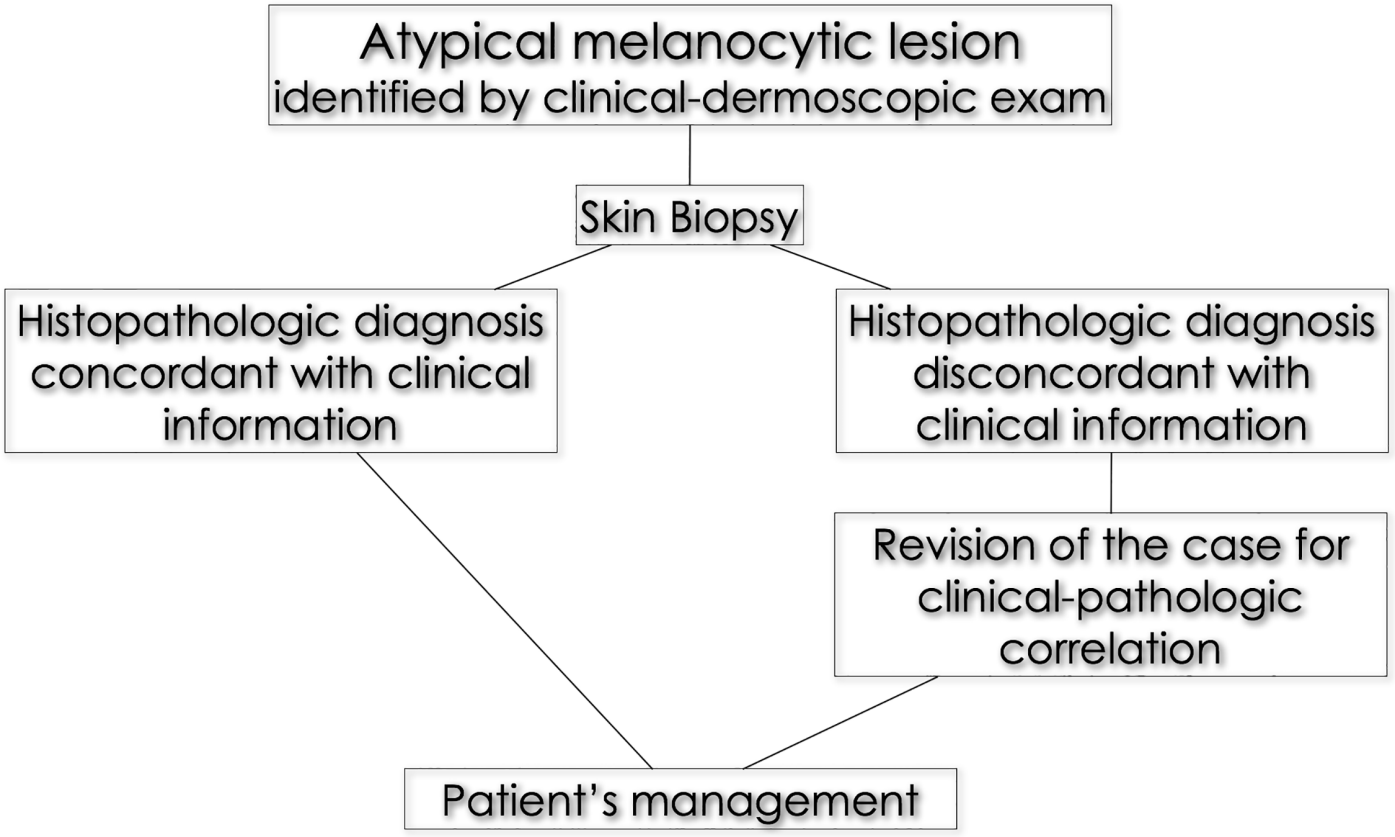
Workflow for the management of atypical melanocytic lesions in our tertiary referral center. When the histopathologic report is rendered, the clinician reviews the case in light of the clinical-dermoscopic pictures. Cases for which a good clinico-pathologic correlation is missing are jointly reviewed by clinicians and pathologists combining all relevant clinical and histopathologic data including clinical-dermoscopic images. A consensus diagnosis is finally reached and the patient treated accordingly.

After the excision of a skin lesion, the clinician fills the referral sheet with anagraphic data, site of the lesion and reports his/her clinical diagnosis. When the histopathologic report is rendered, the clinician reviews the case in light of the clinical-dermoscopic pictures. Cases for which a good clinico-pathologic correlation is missing are jointly reviewed by the referral clinicians and the referral dermatopathologist combining all relevant clinical and histologic data including clinical-dermoscopic images and a picture selection of histopathologic slides. A final consensus diagnosis is then reached in light of the case discussion (**Fig 1**).

## Results

In the time-frame of this study, 3600 skin biopsies were performed, including 709 melanomas, 1167 basal cell carcinomas, 388 squamous cell carcinomas, 596 nevi, 596 other benign lesions and malignant lesions. Five hundred ninty six benign and malignant lesions included 314 seborrheic keratosis, 32 sebaceous cysts, 55 pyogenic granulomas, 71 dermatofibromas, 25 clear cell acanthomas, 12 dermatofibrosarcoma protuberans, 11 Merkel cell carcinomas, 23 adnexal tumors, 15 atypical fibroxantomas, 12 eccrine poromas and 26 other not classified tumors. One hundred fifty-eight lesions out of 3600 (4.4%) were jointly reviewed during the clinic-pathologic meeting, including melanocytic and non-melanocytic tumors, skin lymphomas and inflammatory skin diseases. If we exclude basal cell carcinomas and squamous cell carcinomas from the analysis since they are not usually controversial lesions, the total cases that are reviewed were 158 out of 2045 (7.7%).

All 158 lesions had a complete set of clinical and dermoscopic images and in 58 (37%) RCM images were also available. Cases included 67 women and 91 men with a mean age of 51.8 years (interquartile range, 3–88 years). Lesions were predominantly located on trunk (73; 46.2%) followed by lower limbs (34; 21,5%), head and neck area (26; 16.4%), upper limbs (15; 9.4%), acral site (7; 4.4%) and nail apparatus (1; 0.6%).

Out of 158 lesions discussed in the consensus meeting, 31 had been initially diagnosed as melanoma (19.6%), 74 junctional or compound nevus (46.8%), 22 Spitz/Reed nevus (13.9%), 2 basal cell carcinoma (1.3%), 4 squamous cell carcinoma (2.5%), 2 mycosis fungoides (1.3%), and 23 other tumors including adnexal neoplasias (14.5%). The two cases of mycosis fungoides were discussed because the provisional first histologic diagnosis was spongiotic dermatitis. However the clinical picture was more suggestive for a mycosis fungoides and thus we did a reappraisal of the cases and a second punch biopsy.

After re-evaluation of all lesions missing a good clinical-pathologic correlation, the first histopathologic diagnosis was changed in 13 cases (8.2% of the 158 reviewed lesions and 7.7% of the 2045 excised lesions, excluding basal cell and squamous cell carcinomas) (**Table 1**); all 13 were melanocytic lesions. The clinical-pathologic meeting allowed changing the histopathologic diagnosis from nevus to melanoma in 12 cases (all intraepidermal or thin lesions with Breslow thickness less than 1 mm) and from melanoma to nevus in one case. The majority of the lesions were located on the trunk (8/13; 61.5%), followed by head/neck (3/13; 23%) and two cases on the limbs (15%).

**Table 1:** List of cases missing a clinical-pathologic correlation: demographic description and reasons for diagnosis re-appraisal.

Case	Gender	Age	Body site	I° histologic diagnosis	II°histologic diagnosis	Reasons for diagnosis reappraisal
1	m	62	head/neck	junctional nevus	melanoma in situ	not committed pathologist
2	m	75	back	junctional nevus	melanoma in situ	not committed pathologist
3	m	71	thorax	combined nevus	melanoma arising on a pre-existing nevus	Clinical picture and age of the patient
4	m	48	thorax	junctional nevus	melanoma in situ	Ambiguous histology, disagreement among Pathologist
5	f	52	back	junctional nevus	melanoma in situ	Clinical picture
6	m	67	back	compound nevus	melanoma arising on a pre-existing nevus	Clinical picture
7	f	44	back	junctional nevus	melanoma in situ	Clinical picture
8	m	46	head/neck	junctional nevus	melanoma 0.5 mm Breslow	punch issue
9	m	57	back	compound nevus	melanoma in situ	Clinical picture
10	m	65	head/neck	junctional nevus	melanoma in situ	not committed pathologist
11	f	62	lower limb	junctional nevus	melanoma in situ	Clinical picture
12	m	32	abdomen	melanoma arising on a pre-existing nevus	compound nevus	long-standing lesion
13	m	32	upper limb	compound nevus	melanoma 0.5 mm Breslow	digital follow-up, growing lesion

**Fig 2:**
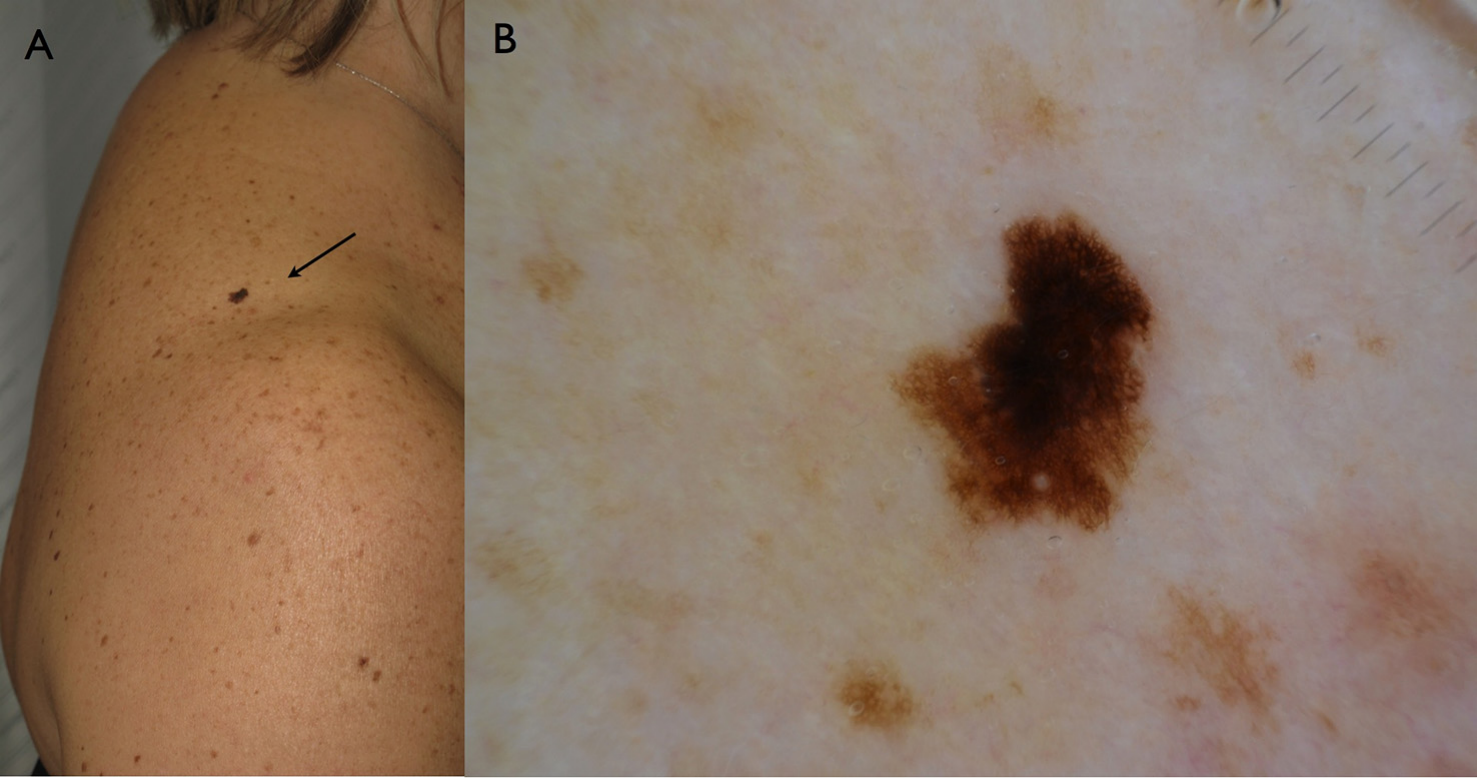
A. Solitary hyperpigmented flat lesion on the shoulder of a 52 years old lady. B. dermoscopy reveals an irregularly shaped lesion with atypical network.

**Fig 3:**
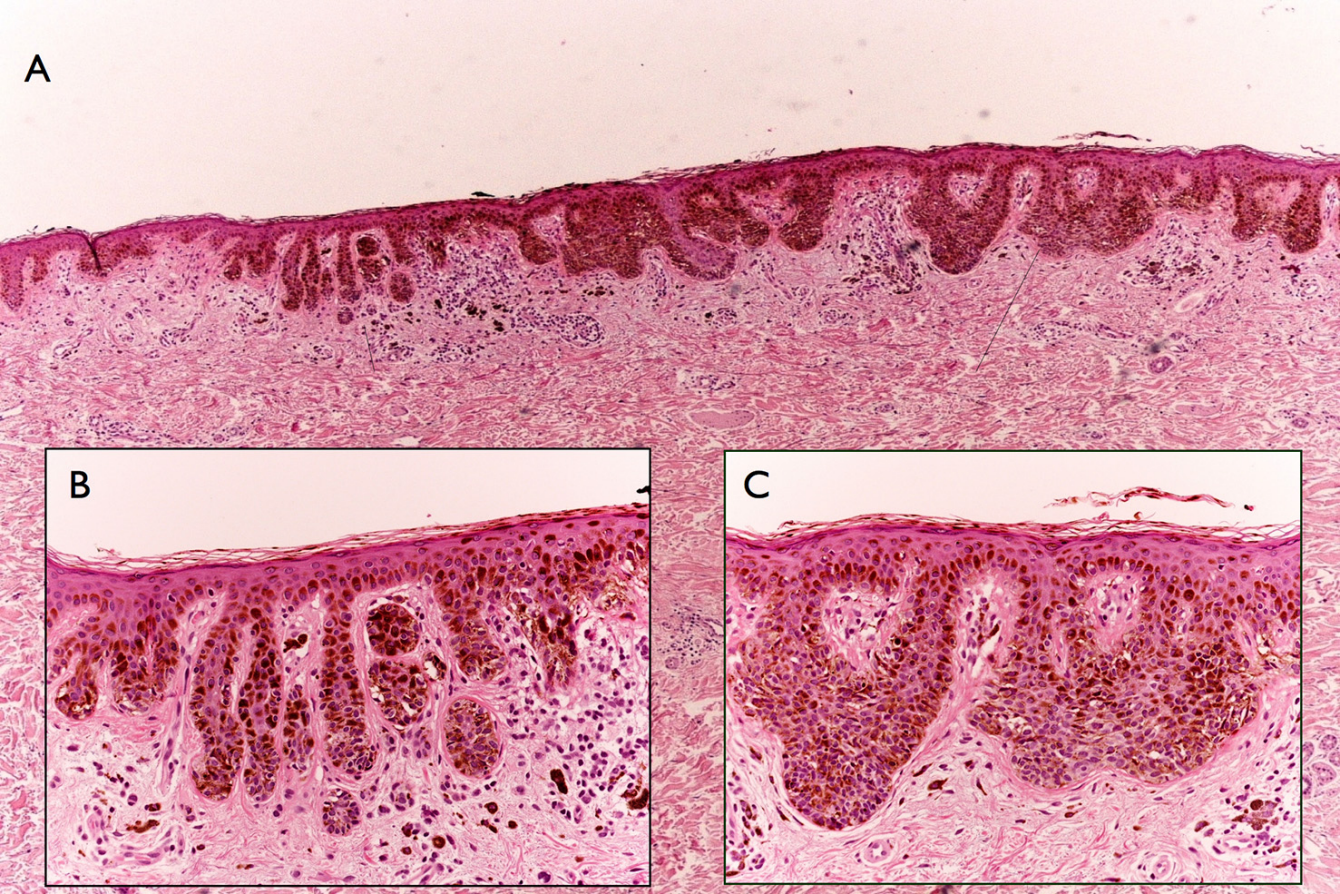
A. On histology, there is a diffuse, lentiginous proliferation made up by pigmented, monomorphous melanocytes. B and C. The cells are cytologically bland and mainly located in the lower epidermis. Pagetoid spread is not a feature.

**Fig 4:**
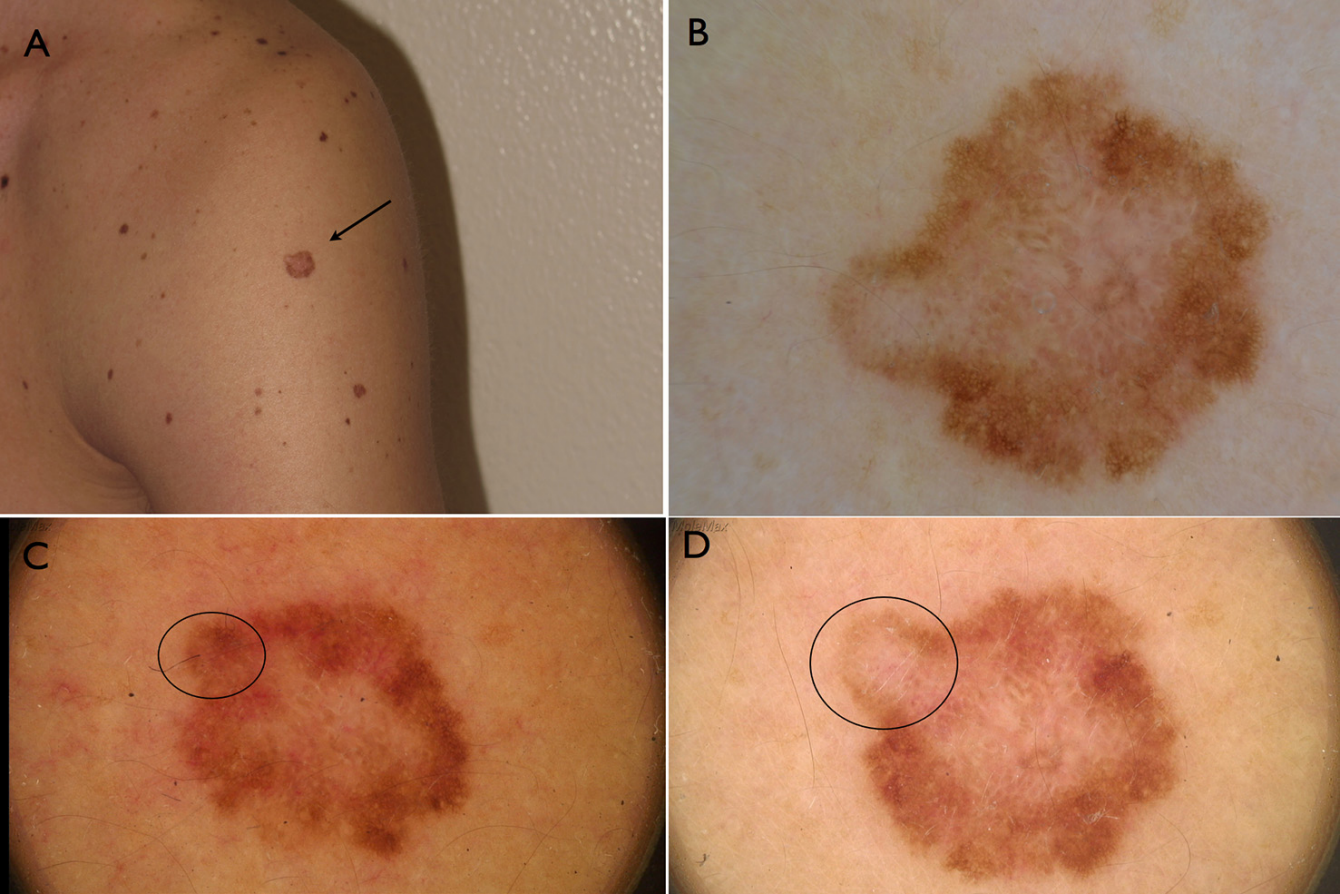
A. Large flat lesion on the arm of a 32 years old man. B. at the time of surgical excision, dermoscopy reveals the presence of asymmetry of color and structure, with pigment network and regressive features suggestive for melanoma. C and D. Digital dermoscopic follow up of the lesion reveals the asymmetric growth of the tumor (circle) over time.

**Fig 5:**
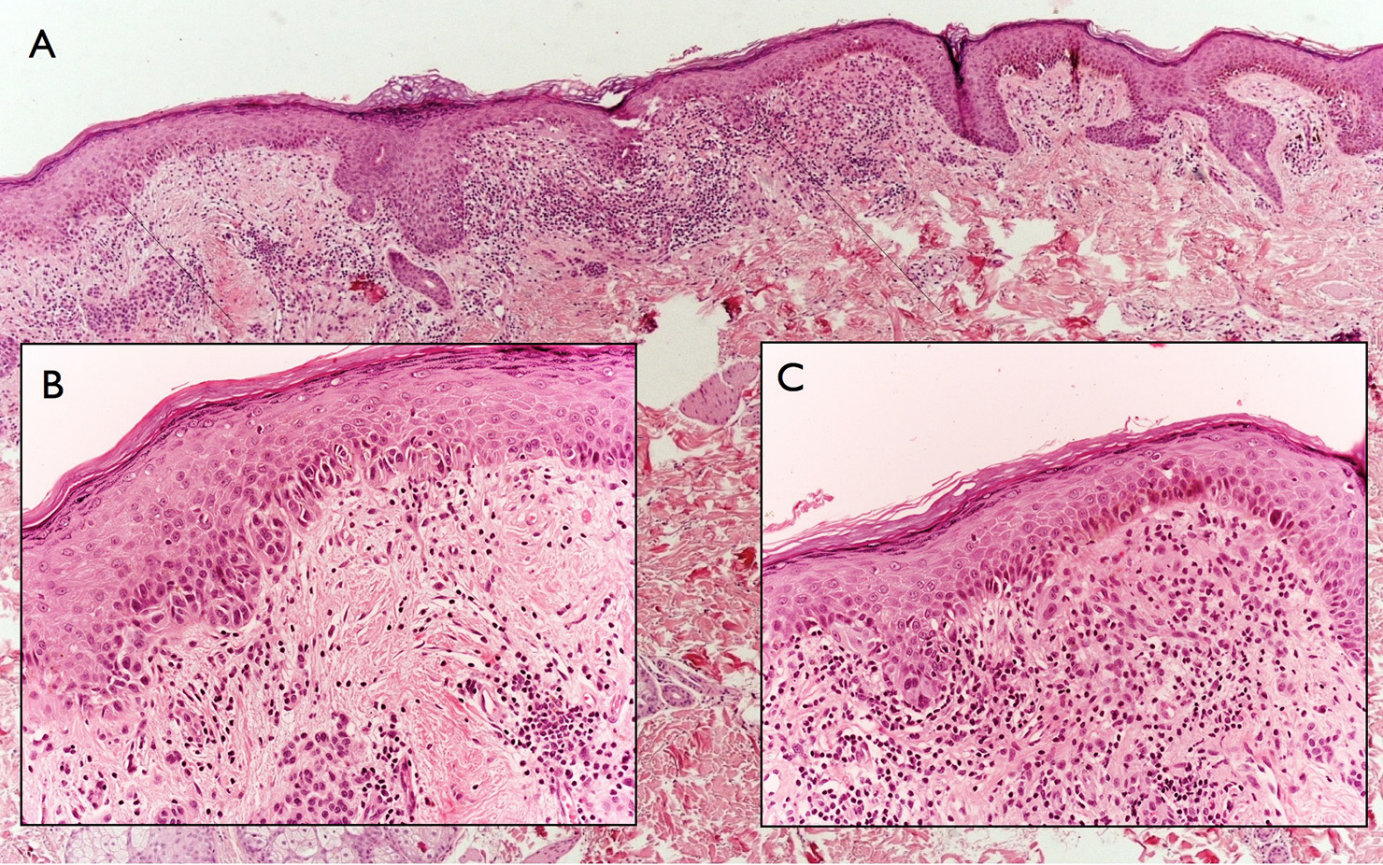
A. Histopathology shows an irregular melanocytic lesion, associated with a slightly thickened epidermis. B and C. Junctional melanocytes are rather pleomorphic while the dermal component is bland and monomorphous. Fibrosis and inflammation are evident in the superficial dermis.

Reasons for diagnosis reappraisal following the consensus meeting were mainly due to the discordant clinical aspect of the lesion. More specifically, in 6 cases despite bland/inconspicuous histopathologic features, the overall clinical picture (i.e. solitary lesion, ugly duckling sign, heavily pigmented lesion in the elderly) strongly supported the diagnosis of melanoma that therefore could not be rendered confidently based solely on the limited set of histopathologic features (**Figs 2 and 3**). In one case the diagnosis was made on a punch biopsy in which the conspicuous lesion size and all the set of histopathologic features were missing (case 8). The evident growing attitude of the lesion documented by digital monitoring was supporting the histopathologic diagnosis of melanoma in one case that had been histopathologically underestimated (case 13) (**Figs 4 and 5**). Conversely, the long-standing history of the lesion (case 12) reported by the patient favored the final diagnosis of an asymmetrical nevus with sclerosis rather than a melanoma arising on a nevus. Four cases were initially under-diagnosed by a general surgical pathologist without formal dermatopathology training. A second opinion was requested in 6 out of 13 cases, confirming the reappraisal of the diagnoses as reported by the referral dermatopathologist of our center.

Following the consensus meeting, all patients were treated according to the final diagnosis, namely, wider surgical excision if needed, and appropriate follow-up program.

## Discussion

In the present study assessing the impact of clinical-pathologic discussion in daily practice, the final diagnosis and subsequent patient management was changed in 8.2% of lesions that were reevaluated at the clinical-pathologic meeting. It is well know that a certain proportion of melanocytic lesions are difficult to interpret even for expert and specifically trained pathologists. Some lesions show an ambiguous combination of morphologic criteria which interpretation carries a high level of subjectivity resulting in a low interobserver agreement even among expert pathologists [2,7]. In such difficult cases, the addition of the clinical information including dermoscopy may help to reach a more confident diagnosis.

Bauer et al.[6] retrospectively analyzed a series of 301 nevi and melanomas. Due to the analysis of dermoscopic images by the pathologist, the final diagnosis was revised in 11 cases (4%), 8 from melanoma to nevus and 3 from nevus to melanoma. Moreover, when pathologists at 2 distinct centers evaluated this series, the addition of dermoscopic images improved concordance between centers from a k value of 0.81 to 0.88. More recently, Ferrara et al.[7] demonstrated that the number of lesions with a consensus diagnosis and the subsequent k statistics were significantly higher when all the clinical information are available, suggesting that the histopathologic diagnosis can be improved by the knowledge of clinical data.

Although several studies clearly demonstrated the increased diagnostic accuracy when clinical-pathologic correlation is performed, they were all conducted retrospectively, and thus, the real impact of this strategy in clinical practice was never assessed before. In our series, a clinical-pathologic discussion was required in 4.4% (158 out of 3600) of all excised lesions and in 0.4% of them (13 out of 3600; all of them being melanocytic lesions) an agreement on the change of the management was made based on the consensus meeting.

This agreement between Clinicians and Histopathologists regarded three main scenarios. First, lesions that were typified by a cytologically bland, lentiginous, junctional, melanocytic proliferation arising in the elderly (9/13). All these lesions were clinically highly suspicious, especially because of their solitary nature and their atypical morphologic features [13–15]. Considering all together clinical and histologic features we reached an agreement toward a cautious management of the case as per melanoma in situ.

The second scenario concerned one lesion showing bland histopathologic features but revealing striking malignant changes during digital monitoring. It is well-known that the analysis of changes over time permits to differentiate those occurring in nevi (growing slowly and symmetrically) from those occurring in melanoma (usually growing faster and asymmetrically)[16–18].

Lastly, our study highlighted the importance of a specific expertise in dermatopathology for diagnosing ambiguous melanocytic lesions [19]. In our series, 3 lesions were initially diagnosed as junctional nevus by the surgical pathologist. The lesions were then revised by the expert dermatopathologist and a diagnosis of melanoma was finally rendered.

Our study has some limitations. By limiting a clinicopathological evaluation to cases with 'discordant' clinical and histopathological data, we did not assess cases in which histopathological diagnosis could be wrong even if in agreement with the clinical data. However, this point is difficult to be addressed since it implies a review of all cases excised in the clinical setting.

By being aware of these diagnostic difficulties, clinicians may actively contribute to achieve a more confident histopathologic diagnosis. A detailed clinical assessment, an improved communication with the dermatopathologist and a cautious interpretation of the final histopathology report are the key points to select those lesions requiring careful clinical-pathologic reevaluation.

In conclusion, our study demonstrated that an integrated approach involving clinicians and pathologists allows improving management of selected patients by shifting from a simply disease-focused management (melanoma *versus* nevus) to a patient-centered approach.
